# Premature mortality due to social and material deprivation in Nova Scotia, Canada

**DOI:** 10.1186/s12939-014-0094-2

**Published:** 2014-10-25

**Authors:** Nathalie Saint-Jacques, Ron Dewar, Yunsong Cui, Louise Parker, Trevor JB Dummer

**Affiliations:** Cancer Care Nova Scotia, Surveillance and Epidemiology Unit, Room 560 Bethune Building, 1276 South Street, Halifax, Nova Scotia B3H 2Y9 Canada; Interdisciplinary PhD program, Dalhousie University, 6299 South Street, Room 314, PO Box 15000, Halifax, Nova Scotia B3H 4R2 Canada; Population Cancer Research Program, Department of Pediatrics, Dalhousie University, 1494 Carlton Street, PO Box 15000, Halifax, Nova Scotia B3H 4R2 Canada; School of Population and Public Health, The University of British Columbia, Room 165-2206 East Mall, Vancouver, British Columbia V6T 1Z3 Canada

**Keywords:** Socioeconomic factors, Premature mortality, Small-area analysis, Deprivation index, Public health surveillance, Health equity

## Abstract

**Introduction:**

Inequalities in health attributable to inequalities in society have long been recognized. Typically, those most privileged experience better health, regardless of universal access to health care. Associations between social and material deprivation and mortality from all causes of death— a measure of population health, have been described for some regions of Canada. This study further examines the link between deprivation and health, focusing on major causes of mortality for both rural and urban populations. In addition, it quantifies the burden of premature mortality attributable to social and material deprivation in a Canadian setting where health care is accessible to all.

**Methods:**

The study included 35,266 premature deaths (1995–2005), grouped into five causes and aggregated over census dissemination areas. Two indices of deprivation (social and material) were derived from six socioeconomic census variables. Premature mortality was modeled as a function of these deprivation indices using Poisson regression.

**Results:**

Premature mortality increased significantly with increasing levels of social and material deprivation. The impact of material deprivation on premature mortality was similar in urban and rural populations, whereas the impact of social deprivation was generally greater in rural populations. There were a doubling in premature mortality for those experiencing a combination of the most extreme levels of material and social deprivation.

**Conclusions:**

Socioeconomic deprivation is an important determinant of health equity and affects every segment of the population. Deprivation accounted for 40% of premature deaths. The 4.3% of the study population living in extreme levels of socioeconomic deprivation experienced a twofold increased risk of dying prematurely. Nationally, this inequitable risk could translate into a significant public health burden.

## Introduction

Inequities in health are entrenched in society, often reflecting disparities in the conditions in which people live, work, and play [1-3]. In 1980’s, Townsend [4] articulated this concept as “deprivation”: “an observable and demonstrable disadvantage relative to the local community or the wider society or nation to which the individual, family or group belong”. Deprivation is, therefore, a measure made *relative* to some privileged group or social norm, a norm which can differ between places and change over time. Townsend distinguished two forms of deprivation: *material* deprivation which relates to the access of goods and conveniences and; s*ocial* deprivation which refers to disadvantages related to social position.

The influences of social and material deprivation on health are many and their magnitude and direction differ between health outcomes [5-10]. Mortality, a measure of population health, is often lower amongst privileged individuals or communities; a pattern observed across and within many countries, including those offering universal health coverage [11-18]. Recent trends for widening socioeconomic inequalities may further increase inequity in mortality rate—in particular, the rate of premature mortality [19-22]. From a societal view point, the cost of premature mortality (PM) can be measured directly through the increased burden of health care or, indirectly through the premature loss of individuals’ contributions to society over their lifetime [23]. PM is thought to be avoidable and, therefore unacceptable [24]. In Canada, the impact of social and material deprivation on PM from all causes varies by geographic area, despite universal access to health care [14]. However, the relationship between social and material deprivation and major causes of premature death, as well as the overall magnitude of their effects, has yet to be reported, either at the national or provincial levels.

Compared to other provinces in Canada, Nova Scotia (NS) has high mortality rates and the second to lowest gross income per capita [25]. Further, NS has a high proportion of rural residents who in general have lower income and may experience a disproportionate burden of material and social deprivation. It is, therefore, an ideal location to examine the links between PM and socioeconomic deprivation. This study evaluates the relationship between social and material deprivation and PM in NS using a recently validated index [26]. It also quantifies the number and proportion of premature deaths directly attributable to socioeconomic deprivation, were the association considered to be causal. The results of this study will inform public health programs and policies aimed at addressing health inequities resulting from socioeconomic disparities.

## Methods

### Deprivation indices

Area-based deprivation indices were developed in the UK [4,27,28] as a tool for investigating socio-economic variations in health and as a surrogate indicator of individual-level socioeconomic status. They have since been modified to reflect the local reality and data availability of various populations around the world [29-37]. For this study, two indices of deprivation were constructed following the methodology detailed by Pampalon and colleagues [38] developed to measure socioeconomic deprivation within a Canadian context. The indices were composed of six variables from the 2001 Canadian census known to have utility as geographic proxies of socioeconomic conditions [21,33,39,40]. For people age 15 years and over, these variables were: the proportion of people with no high school diploma, the individual average income, the employment rate, the proportion of separated, divorced or widowed, the proportion of single-parent families (lone parent), and the proportion of persons living alone. The first three indicators reflect the material dimension of deprivation; the others reflect its social aspect. All variables, with the exception of the proportion of single-parent families, were adjusted to the age and sex structure of the 2001 NS population aged 15 years and older, using indirect standardization [41]. Transformations (log– for continuous variables, arcsin of square root– for proportional indicators) were applied to normalize the indicators. Variables were combined using a Principal Component Analysis (PCA), a standard factorial approach that recognizes the interlinked nature of variables by accounting for their correlation and co-variation [42]. Following a varimax rotation, two independent components with eigenvalues exceeding 1.0 were retained for interpretation. These components were defined as ‘material index’ and ‘social index’ of deprivation, respectively.

The indices were constructed at the smallest unit of census geography, the dissemination area (DA), which comprises generally a population of 400–700 persons but which can be as low as 40 persons in rural NS and as high as 3,600 in urban NS. DAs were defined as urban when in proximity to a census metropolitan area with a population density of 400 or more people per square kilometer as outlined in Du Plessis et al. [43]. In the 2001 census, NS was covered by 515 urban and 771 rural DAs (excluding First Nations reserves, for which details of population and census variables were incomplete). PCA produced factor scores for all 1,286 DAs. The DAs were ranked according to their factor scores and grouped into weighted population quintiles, one distinct set of quintiles for each level of geography (i.e. urban, rural, NS as a whole). This was done to account for differences in the range of factor scores by level of geography. In all instances, quintile 1 (Q1) represented the most privileged segment of the population and quintile 5 (Q5), the least. This process was carried out separately for each of the deprivation indices.

### Premature mortality (PM)

Mortality data coded ICD-9 (1995 – 1999) or ICD-10 (2000–2005) for NS residents who died between 1995–2005 were obtained from NS Vital Statistics. Deaths were grouped into five categories: cancer (ICD9—140-208; ICD10—C00-C97), circulatory system (ICD9—390-459; ICD10—I00-I99), external causes (ICD9—800-999; ICD10—V01-Y98), other causes and all causes. PM was defined as deaths occurring prior to the median age at death (75 for men, 81 for women) observed in this period. Age 75 is often used as a fixed upper threshold age for the calculation of PM, however, an older cut-off was used for females as to reflect their longer life expectancy. Residential postal code at death was used to assign each death to a DA using the Statistics Canada Postal Code Conversion program (PCCF+, version 5G). There were 87,484 deaths over the 11-year period. Of these, 74,610 deaths had postal code information and 73,088 (98%) were successfully geo-referenced to a DA. Two percent of deaths occurred before age 15 and these were excluded. PM rates were based on a total of 35,266 premature deaths and calculated using the 2001 NS population aged 15 years plus, obtained from Statistics Canada. An aggregated dataset of premature death counts was used to estimate PM rates for each quintile of material and social deprivation, from the most (Q1) to the least privileged (Q5), and for groups experiencing extreme socioeconomic conditions, including those materially and socially most privileged (Q1material-Q1social; Q1 & Q1) which accounted for 7.0% of the NS population aged 15 years and older, and those materially and socially least privileged (Q5material-Q5social; Q5 & Q5) which accounted 4.3%.

### Analytical method

The influence of deprivation on PM was modeled with Poisson regression. In Model 1, quintiles of material and social deprivation were used as categorical variables and so accounted for the main effects of the two indices. In Model 2, for every combination of material and social deprivation quintiles, mean material and social deprivation scores were calculated and modeled with their interaction with population location (urban/rural). PM rate ratios and absolute excess mortality were also examined. Rate ratios (rate for the least privileged (Q5material-Q5social) divided by the rate for the most privileged (Q1material-Q1social), and corresponding 95% confidence intervals were derived from a Poisson regression model. The excess mortality measure estimated the absolute number of premature deaths for any subgroup that could be potentially avoided if the whole population had the same PM rate as that of the most privileged group. Data analyses were performed using SAS 9.1 and R 2.13.0. The study received ethics approval from Capital Health and IWK Health Centre Research Ethics Boards.

## Results

### Socioeconomic deprivation

The PCA identified two main components, together accounting for 67% of the variation associated with the six indicators. The first component reflected material deprivation, with high loadings for education (0.89), income (−0.84) and employment (−0.62); the second component reflected social aspects, with high loadings of the proportion of separated, divorced or widowed (0.89), the proportion of persons living alone (0.78) and of single-parent families (0.64). The population profile by quintile of material and social deprivation is presented in Table 1. Of particular interest is the comparison between the least and most privileged groups (Q5material-Q5social vs. Q1material-Q1social, respectively) which shows that the former had 4.1 times higher proportion of people without a high school diploma (e.g. 47.6% vs. 11.5%); 1.7 times lower employment rates; 3.1 times higher number of people living alone; 2.4 times higher number of people identified as separated, divorced or widowed; and 6.1 times higher number of single-parent families. In addition, the least materially and socially privileged people earned less than half the income of the most privileged ($16.7 K vs. $40.5 K). These differences between the least and most materially and socially privileged groups were observed in both rural and urban NS, but were generally greater in urban populations (Table 1). The exception was for employment rate for which the gap between the most and least privileged group was greater in rural NS (Table 1).

**Table 1:** **Characteristics of study population age 15 years and older, by quintile of material and social deprivation, and those of the most and least materially and socially privileged population groups, Nova Scotia** ^a^

**Deprivation quintile**		**No high school diploma %**	**Employment rate %**	**Individual average income $**	**Living alone %**	**Separated divorced widowed %**	**Lone parent %**
**Material**							
privileged	Q1	15.5	63.0	34,224	14.1	19.7	12.4
	Q2	24.1	59.2	26,536	10.9	17.6	15.3
	Q3	31.9	54.8	23,785	9.8	17.7	16.3
	Q4	38.5	50.4	21,264	10.0	18.7	17.7
deprived	Q5	50.4	42.8	18,791	9.0	18.5	22.8
**Social**							
privileged	Q1	27.0	58.4	30,096	5.0	11.5	7.5
	Q2	34.1	54.7	25,113	7.1	14.8	12.4
	Q3	36.9	52.3	23,334	8.6	17.4	15.9
	Q4	32.2	51.5	24,406	12.2	20.2	20.0
deprived	Q5	31.2	52.8	22,274	19.9	27.5	27.7
**ALL of Nova Scotia: Material and social**							
most privileged^b^	Q1 & Q 1	11.5	66.5	40,498	4.0	9.9	6.8
least privileged^c^	Q5 & Q5	47.6	39.5	16,650	12.4	23.7	41.5
**RURAL Nova Scotia: Material and social**							
most privileged	Q1 & Q 1	17.3	65.8	33,345	4.2	10.5	6.3
least privileged	Q5 & Q5	47.3	39.6	17,410	9.8	20.8	34.2
**URBAN Nova Scotia: Material and social**							
most privileged	Q1 & Q 1	7.8	66.1	47,091	4.9	9.9	7.8
least privileged	Q5 & Q5	44.7	45.7	17,009	17.2	27.8	44.6

^a^Source: 2001 Census of Canada.

^b^Include those people who are most materially and socially privileged, Q1 & Q1.

^c^Include those people who are least materially and socially privileged, Q5 & Q5.

### Socioeconomic deprivation and premature mortality

**Figure 1:**
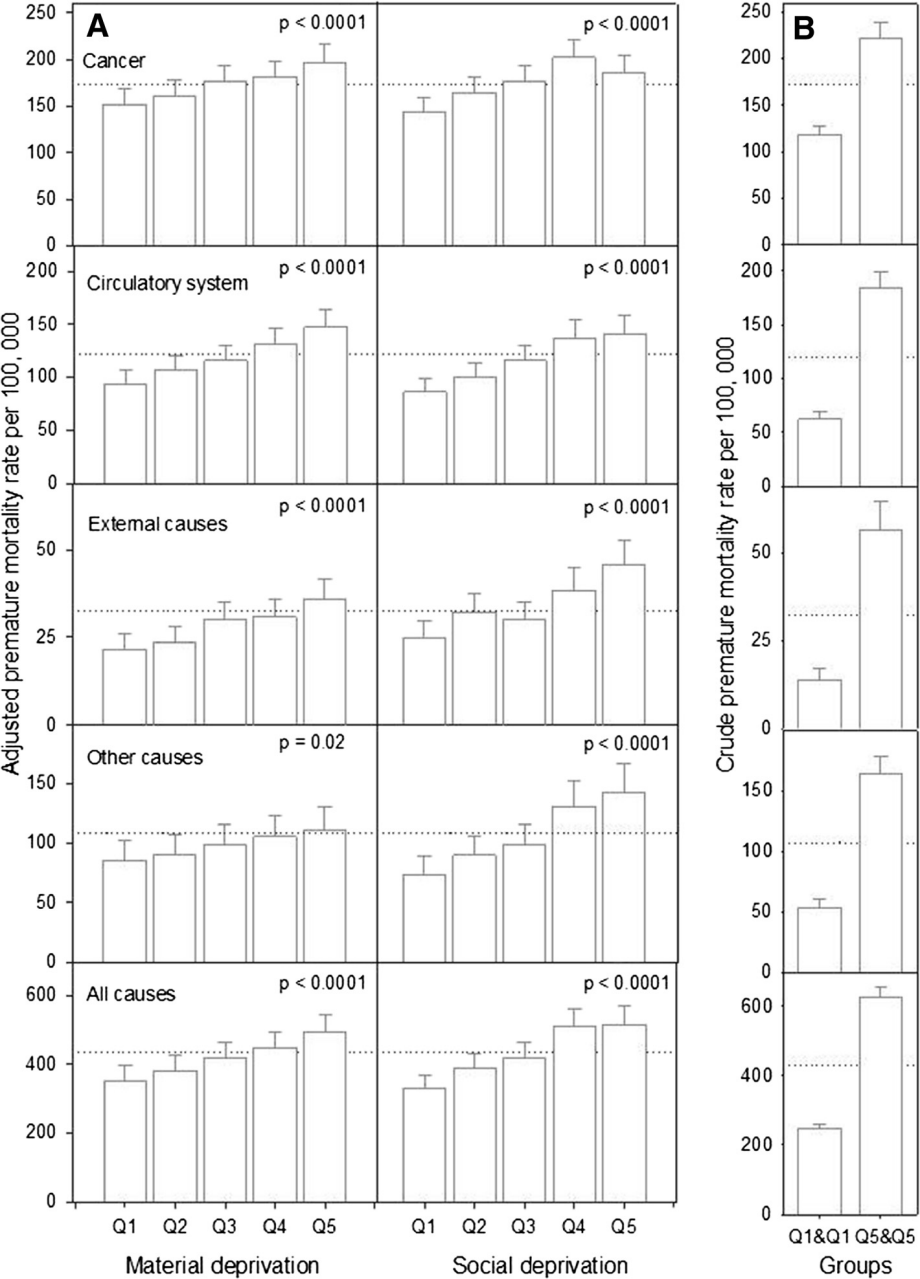
**Adjusted (panel A) and crude (panel B) premature mortality rate for population age 15 years and older, by quintile of material and social deprivation and causes of death, Nova Scotia 1995–2005.** Dotted line represents the adjusted **(panel A)** and crude **(panel B)** premature mortality death rates for Nova Scotia. P-values are from one-tailed test.

Of the 35,266 premature deaths included in the study, 14,054 (40%) were attributed to cancer, 9,793 (28%) to disease of the circulatory system, 2,646 (8%) to external causes and 8,773 (25%) to other causes (Table 2). The total number of premature deaths was greater in rural than urban NS (20,506 vs 14,752) but crude PM rates did not differ significantly between urban and rural areas, with the exception of other causes mortality for which the rate was higher in urban populations (Table 2). Both crude (Table 2) and adjusted PM rates (Model 1, Figure 1) increased monotonically with increasing levels of material and social deprivation. For social deprivation, these rates showed higher mortality in Q4 for cancer and all causes mortality.

**Table 2:** **Population counts, premature death counts, crude premature death rates** ^**a**^ **and associated 95% confidence interval by geographic areas** ^**b**^ **, quintiles of social and material deprivation, and major causes of mortality, Nova Scotia 1995-2005**

		**Cancer**				**Circulatory system**				**External causes**				**Other causes**				**All causes**			
				**95% CI**				**95% CI**				**95% CI**				**95% CI**				**95% CI**	
**Geographic area**	**Population** ^**c**^ **Count**	**Count**	**Rate**	**from**	**to**	**Count**	**Rate**	**from**	**to**	**Count**	**Rate**	**from**	**to**	**Count**	**Rate**	**from**	**to**	**Count**	**Rate**	**from**	**to**
NS	742,580	14,054	172.1	169.2	174.9	9,793	119.9	117.5	122.3	2,646	32.4	31.2	33.7	8,773	107.4	105.2	109.7	35,266	431.7	427.2	436.3
Urban	309,660	5,880	172.6	168.2	177.1	3,991	117.2	113.6	120.9	1,060	31.1	29.3	33.1	3,821	112.2	108.6	115.8	14,752	433.1	426.1	440.1
Rural	432,920	8,174	171.6	167.9	175.4	5,797	121.7	118.6	124.9	1,585	33.3	31.7	35	4,950	103.9	101.1	106.9	20,506	430.6	424.7	436.5
**Material**																					
Q1	148,290	2,403	147.3	141.5	153.3	1,504	92.2	87.6	97.0	398	24.4	22.1	26.9	1,522	93.3	88.7	98.1	5,827	357.2	348.1	366.5
Q2	148,365	2,577	157.9	151.9	164.1	1,712	104.9	100.0	110.0	427	26.2	23.7	28.8	1,575	96.5	91.8	101.4	6,291	385.5	376.0	395.1
Q3	148,535	2,838	173.7	167.4	180.2	1,879	115.0	109.9	120.3	551	33.7	31.0	36.7	1,713	104.8	99.9	109.9	6,981	427.3	417.3	437.4
Q4	148,685	2,959	180.9	174.5	187.6	2,184	133.5	128.0	139.3	577	35.3	32.5	38.3	1,893	115.7	110.6	121.1	7,613	465.5	455.1	476.1
Q5	148,705	3,277	200.3	193.5	207.3	2,514	153.7	147.7	159.8	693	42.4	39.3	45.6	2,070	126.5	121.2	132.1	8,554	522.9	511.9	534.1
**Social**																					
Q1	147,870	2,221	136.5	130.9	142.3	1,343	82.6	78.2	87.1	351	21.6	19.4	24.0	1,154	70.9	66.9	75.2	5,069	311.6	303.1	320.3
Q2	148,755	2,684	164.0	157.9	170.4	1,727	105.5	100.6	110.6	509	31.1	28.5	33.9	1,489	91.0	86.4	95.7	6,409	391.7	382.1	401.4
Q3	148,250	2,893	177.4	171.0	184.0	2,017	123.7	118.3	129.2	486	29.8	27.2	32.6	1,643	100.8	95.9	105.7	7,039	431.6	421.6	441.8
Q4	148,805	3,283	200.6	193.8	207.5	2,344	143.2	137.5	149.1	601	36.7	33.8	39.8	2,153	131.5	126.0	137.2	8,381	512.0	501.1	523.1
Q5	148,900	2,973	181.5	175.0	188.2	2,362	144.2	138.5	150.1	699	42.7	39.6	46.0	2,334	142.5	136.8	148.4	8,368	510.9	500.0	522.0
**Material and Social**																					
Q1 & Q1	51,985	678	118.6	109.8	127.8	358	62.6	56.3	69.4	79	13.8	10.9	17.2	307	53.7	47.8	60	1,422	248.7	235.9	261.9
Q5 & Q5	31,830	777	221.9	206.6	238.1	645	184.2	170.3	199	198	56.6	48.9	65	574	163.9	150.8	177.9	3,194	626.6	600.7	653.4

^a^Rates per 100,000 people.

^b^A total of 8 deaths could not be assigned to a specific urban or rural area.

^c^2001 Canadian census population, 15 years and older.

**Figure 2:**
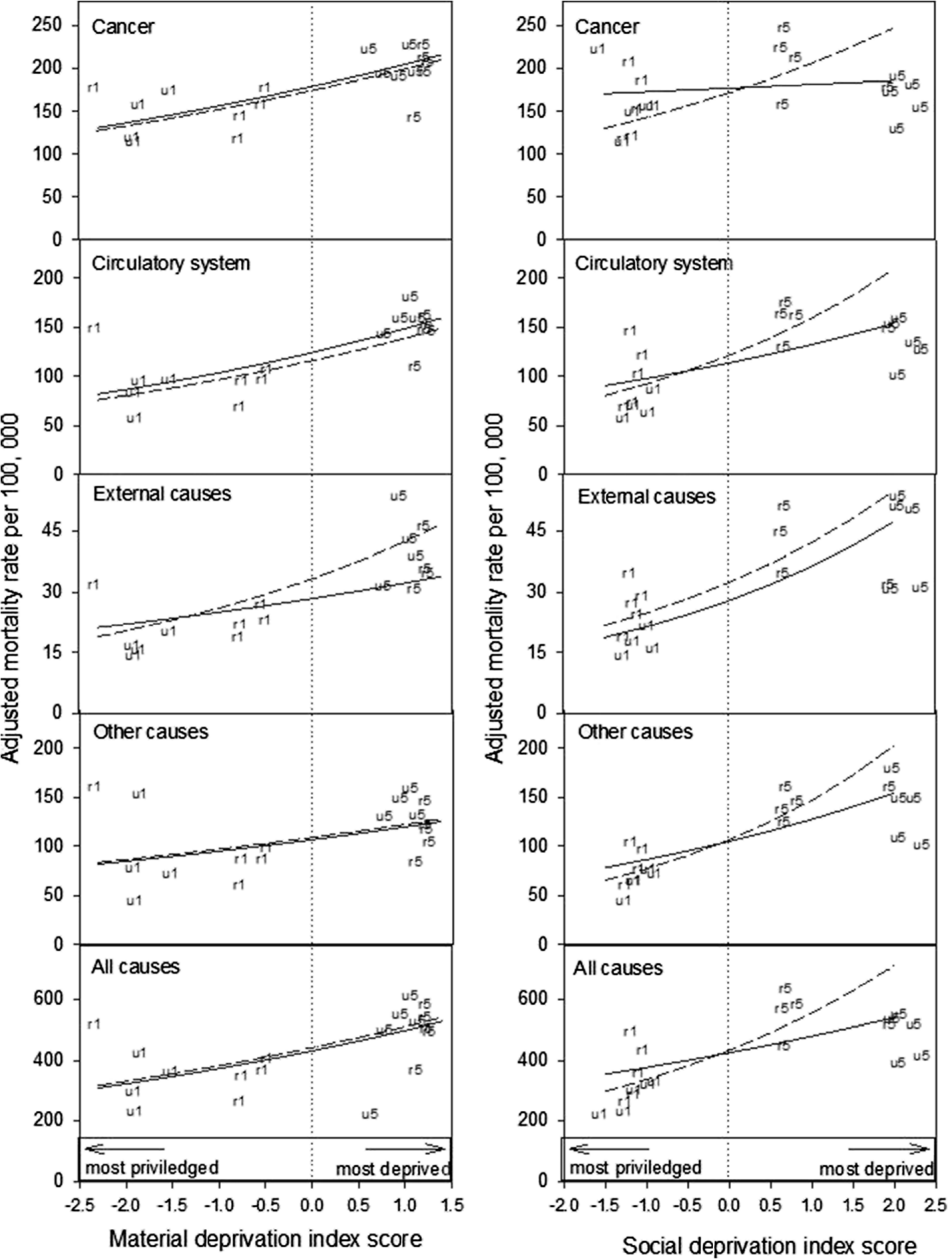
**The relationship between material (left panel) and social (right panel) deprivation index scores and premature mortality rate adjusted for geographic area (urban, rural) and the other form of deprivation, Nova Scotia 1995–2005.** The solid and dashed lines indicate Model 2 predictions for urban and rural populations aged 15 years and older, respectively. For illustrative purposes the mean material and social deprivation scores for the most (urban: u1; rural: r1) and least privileged (urban: u5; rural: r5) groups are shown. The dotted line represents the average population scores.

Crude rate ratios (RRs) in PM for those in the most and least privileged population groups are presented in Table 3. For all causes, the PM rate for NS was 2.5 times higher in people experiencing a combination of the most extreme conditions of material and social deprivation relative to the most privileged (Table 3). PM due to cancer, diseases of the circulatory system, external causes and other causes was 1.9, 2.9, 4.1 and 3.1 times higher respectively in the least compared to the most privileged groups (Table 3).Non-significant differences in RR were observed between urban and rural populations, with RR in urban being slightly higher. Figure 2 shows the relationship between the mean material and social deprivation scores and PM, adjusting for interacting material, social and urban/rural effects (Model 2). Table 4 shows the predicted percentage change in PM corresponding to a change of one quintile level. Again, a significant increase in PM for both major and all causes of death was observed with increasing material and social deprivation (Figure 2; Table 4). Material deprivation had a similar influence upon PM rates in urban and rural populations (Figure 2; Table 4). The exception was PM due to external causes for which an increase in material deprivation scores equivalent to one quintile was associated with a 17% increase in PM among those living in rural areas (Table 4) compared to a 7.7% increase in PM rate for those living in urban areas. The influence of social deprivation upon PM rates was also significant for major and all causes mortality and was generally of larger magnitude for rural populations (Figure 2; Table 4). An increase in social deprivation of one quintile was associated with an increase of 14%, 21%, 25% and 19% in PM, due respectively to cancer, circulatory system, other causes, and all causes of death, among rural populations. In contrast, an increase in social deprivation equivalent to one quintile was associated with significant, but lower comparative increases of 7.8%, 11%, 15% and 8.8% in PM among those living in urban areas. The exception to this pattern was external causes, for which an increase in social deprivation of one quintile resulted in a comparable increase in PM rate (20%; Table 4) in both rural and urban populations. However, irrespective of social deprivation, rural populations had a 16% higher risk of dying prematurely due to external causes than urban populations.

**Table 3:** **Rate ratio in premature mortality for the most and least materially and socially privileged population groups (Q5 & Q5 vs Q1 & Q1), Nova Scotia 1995-2005**

	**Nova Scotia**			**Urban**			**Rural**		
		**95% CI** ^**a**^			**95% CI**		**RR**	**95% CI**	
	**RR**	**from**	**to**	**RR**	**from**	**to**		**from**	**to**
Cancer	1.9	1.7	2.1	1.7	1.4	2.0	1.8	1.6	2.1
Circulatory system	2.9	2.6	3.3	2.8	2.2	3.4	2.3	2.0	2.7
External causes	4.1	3.2	5.3	3.8	2.5	5.6	3.2	2.4	4.3
Other causes	3.1	2.7	3.5	3.3	2.6	4.2	2.4	2.0	2.9
All causes	2.5	2.4	2.7	2.4	2.1	2.7	2.2	2.0	2.4

^a^95% confidence interval.

**Table 4:** **Percent change in premature mortality (PM) associated with social and material deprivation by cause of death** ^**a**^ **, Nova Scotia 1995-2005**

**Cause of death**	**Effect**	**% change in PM per quintile** ^**b**^ **URBAN**	**% change in PM per quintile RURAL**	**Chi-square**	**Pr > ChiSq** ^**c**^
Cancer	urban^d^			0.8	0.37
	material	9.9	9.9	48.2	< 0.001
	urban:material			.	> 0.05
	social	7.8	14	37.3	< 0.001
	urban:social			16.6	< 0.001
Circulatory system	urban			1.64	0.2
	material	14	14	52.7	< 0.001
	urban:material			.	> 0.05
	social	11	21	51.8	< 0.001
	urban:social			6.03	0.01
External causes	urban			6.5	0.01
	material	7.7	17	30.9	< 0.001
	urban:material			3.9	0.05
	social	20	20	81.8	< 0.001
	urban:social			.	> 0.05
Other causes	urban			0.07	0.8
	material	8.4	8.4	16.5	< 0.001
	urban:material			.	> 0.05
	social	15	25	52.2	< 0.001
	urban:social			4.97	0.03
All causes	urban			0.21	0.65
	material	11	11	52.3	< 0.001
	urban:material			.	> 0.05
	social	8.8	19	65.2	< 0.001
	urban:social			10.3	0

^a^Based on Poisson regression Model 2 of material and social deprivation and potential interaction with urban and rural residence.

^b^Assuming that on a continuous scale, a 0.7 change in PCA score equates approximately to a change from one quintile level to the next.

^c^One-tailed test.

^d^Where 'urban' is defined as rural = 0; urban = 1 in Poisson regression model.

Considering the distribution of mean material and social deprivation scores for urban and rural populations, there is a greater distribution gap between the most and least privileged in urban NS. With regards to material wealth, those most privileged in urban areas (i.e. u1 [Q1 urban]; Figure 2) were comparatively better off than their rural counterpart (r1 [Q1 rural]; Figure 2). With regards to social wealth, those least privileged in urban areas (i.e. u5 [Q5 urban; Figure 2) were comparatively worse off than their rural counterparts (r5 [Q5 rural]; Figure 2). These differences observed between comparable quintiles in urban and rural populations, were not associated with a significant health advantage in those most materially privileged; nor with a significant health disadvantage in those most socially deprived.

### Population attributable risk

Mortality attributable to variability in death rates across quintiles of material and social deprivation for urban and rural NS is presented in Table 5. Material deprivation alone may have accounted for 7,245 premature deaths over an 11 year-period in NS (3,825 in urban; 3,420 in rural). The independent effect of social deprivation was even more pronounced, accounting for 9,993 premature deaths (5,032 in urban; 4,961 in rural). Over an 11 year-period, 14,693 premature deaths (6,878 in urban; 7,815 in rural) could have been avoided if material and social disparity did not exist (i.e. if all population quintiles had the same mortality rate as Q1, the most privileged). Overall, the combined effect of material and social deprivation accounted for nearly half of all premature deaths recorded in urban populations; and over a third of those recorded in rural populations (Tables 2 and 5). These proportions varied by cause of death, ranging from 31% (cancer), 43% (circulatory system), 44% (external causes) and 42% (other causes) in rural areas; and from 34% (cancer), 51% (circulatory disease), 54% (external causes) and 60% (other causes) in urban areas (Tables 2 and 5). For NS as a whole, the combined effect of material and social deprivation accounted for 42% of all premature deaths.

**Table 5:** **Excess premature deaths** ^**a**^ **due to the independent and combined effect of material and social deprivation, by cause of death, urban and rural Nova Scotia 1995-2005**

**URBAN NOVA SCOTIA**						
**Quintile**		**Cancer**	**Circulatory system**	**External causes**	**Other causes**	**All causes**
**Independent effect of material deprivation**						
	Q1	REF	REF	REF	REF	REF
	Q2	284	208	34	272	798
	Q3	218	158	23	114	513
	Q4	335	282	88	226	932
	Q5	484	549	130	420	1,583
**Total:**		**1,321**	**1,196**	**276**	**1,031**	**3,825**
**Independent effect of social deprivation**						
	Q1	REF	REF	REF	REF	REF
	Q2	217	231	66	232	745
	Q3	390	508	93	486	1477
	Q4	315	478	144	502	1440
	Q5	187	462	192	529	1,371
**Total:**		**1,110**	**1,679**	**494**	**1,749**	**5,032**
**Material and Social deprivation combined** ^**b**^						
**Total:**		**1,987**	**2,024**	**576**	**2,294**	**6,878**
**RURAL NOVA SCOTIA**						
**Quintile**		**Cancer**	**Circulatory system**	**External causes**	**Other causes**	**All causes**
**Independent effect of material deprivation**						
	Q1	REF	REF	REF	REF	REF
	Q2	28	56	73	−33	124
	Q3	224	232	92	116	663
	Q4	474	453	128	265	1,321
	Q5	464	443	180	225	1,311
**Total:**		**1,190**	**1,185**	**472**	**573**	**3,420**
**Independent effect of social deprivation**						
	Q1	REF	REF	REF	REF	REF
	Q2	87	143	47	209	486
	Q3	396	364	131	275	1,166
	Q4	363	386	51	311	1,110
	Q5	634	650	203	712	2,199
**Total:**		**1,480**	**1,543**	**431**	**1,507**	**4,961**
**Material and Social deprivation combined** ^**b**^						
**Total:**		**2,569**	**2,493**	**692**	**2,062**	**7,815**

^a^Number of deaths that would be avoided if all Nova Scotians had the same premature mortality rate as those that are most privileged.

^b^These counts exclude deaths solely due to material or social deprivation.

## Discussion

### Summary of findings

The study revealed substantial inequalities in socioeconomic conditions in NS, Canada. Similar disparities, although of varying magnitude, have been observed for other regions of the country [14]. For example, in British Columbia the ratios of the least to the most privileged persons were 2.5, 1.4, and 5.3 for the proportion of people without high school diploma, lower employment rate and number of single-parent families; compared to 4.1, 1.7, and 6.1 for Nova Scotia, respectively [14]. While these data indicate greater discrepancies between the ‘rich and the poor’ in NS, the gap in average income between the least and most privileged groups was greater in the larger metropolitan areas of both Toronto ($32.8 K) and Vancouver ($28.9 K) compared to Nova Scotia as a whole ($23.8 K).

Inequalities in mortality were not confined to differences between the ‘rich’ and the ‘poor’ or between the most and least socially or materially deprived, but rather, were observed over the entire socioeconomic spectrum, thus affecting every segment of the population. PM rates decreased monotonically from the most to the least disadvantaged quintile for both major and all causes mortality, with the exception of cancer and all causes mortality for which social deprivation resulted in higher PM rates in Q4. Socioeconomic inequalities were associated with more than a doubling in PM rates (i.e. 2.5 time higher) for the approximate 32,000 Nova Scotians (i.e. 4.3% of the population) experiencing a combination of the most extreme levels of material and social deprivation. Inequalities of similar magnitude have also been reported for Canada as a whole [14]) and Scotland [17]. However, in Scotland, the ratio in PM rate between the most and least privileged, increased from 2.2 in 1981 to 4.3 in 2001 for all causes premature deaths. This widening gap was attributed to a sharp decline in PM in those most privileged at a time of increased PM in those most deprived.

The impact of material deprivation on PM was similar for urban and rural populations, whereas the impact of social deprivation on PM rates was significantly higher for those living in rural areas. The exception was PM due to external causes, which was higher in the most materially deprived rural populations and for which the impact of social deprivation on PM rate were similar in urban and rural populations. The mechanisms contributing to these overall differences are not well understood. With regards to external causes, some studies have reported increased mortality due to external causes with increasing material and social deprivation [44]; others have reported higher mortality due to external causes in rural populations [45,46]; but few have examined the impact of the interaction between urban and rural status, socioeconomic indices and external causes of PM.

This study showed that about 40% (14,696 deaths) of premature deaths over an 11 year-period were attributable to socioeconomic inequalities and thus, potentially avoidable. Of these, more than half were associated with social deprivation alone, a factor seldom accounted for in estimates of health risk in Canada. Due to varying study methodologies and limited research reporting on social disparities in premature mortality, it is difficult to compare these results to other studies. Nonetheless, a recent study indicates that up to 30% of excess deaths (all deaths) reported in sixteen European cities could be attributable to socioeconomic disparities [18]. This figure is somewhat lower than that reported here, but may reflect a greater impact of socioeconomic inequalities on premature mortality in comparison to its impact on all deaths. Thus, the magnitude of the burden of PM due to social and material inequalities has far-reaching implications worldwide. Inequalities are undesirable; they affect everyone in terms of loss of potentially productive members of society, and represent added costs for the health care system and public sector [13,47].

### Strengths and limitations

This study was based on 11 years of provincial vital statistics data of which 98% was successfully geo-referenced, enabling deaths to be linked to census-derived deprivation scores. Other strengths include the use of validated composite measures of deprivation [26], which provide a more complete representation of the variability in deprivation relevant to health than do single indicator variables such as income [42,48,49]. In addition, the weight assigned to each variable included in the construction of the material and social indices of deprivation is determined based on the correlation structure that exists among the variables at the geographic level of interest, rather than being determined *a priori* [38].

A limitation of this study is the lower population densities of rural areas which can result in unstable modeled results [50-52]. Also, DAs can cover larger areas in rural NS, possibly resulting in more heterogeneous population profiles. In addition, as demonstrated earlier, the distribution in material and social wealth varied between urban and rural populations. Each of these factors could have reduced the estimated inequalities in PM rates due to the social and material deprivation in rural populations. A second limitation is that area-based indices can be prone to ecological fallacy when inferences are generalized to the individual level [53]. They are also affected by the modifiable areas unit problem (MAUP), which affects the inference of the results from one scale of observation to another [53]. Third, this study did not account for spatial dependency between DAs. Spatially correlated random effect terms are often used to account for this dependency; however, data provided for the study was aggregated by quintile of social and material deprivation and urban/rural regions and so did not permit such an analysis. Failure to account for spatial dependency may have artificially narrowed the confidence intervals for the *β* coefficients and resulted in an underestimation of the type I error rate. Finally, when calculating a population attributable fraction one assumes a causal relationship between the risk factors and health outcome of interest and independence of the considered risk factors from other factors that influence risk [54]. However, it is unlikely that factors contributing to social and material deprivations are completely independent of other factors linked to PM, thus resulting in a possible overestimation in the overall attributable fraction.

### Local and global perspective

Overall findings of a pervasive impact of socioeconomic deprivation on PM rates in NS are consistent with findings reported in other regions of Canada as well as in the United Kingdom, United States, Australia and elsewhere [1,15,20,37,38,47,55,56]. Poor health outcome was not confined to the most disadvantaged. Socioeconomic inequity affected everyone; a pattern highlighted by the World Health Organization (WHO) not only for the most disadvantaged countries, but for countries of all income levels [24]. The estimated twofold difference in PM rate between the least and most privileged population segments of NS is comparable to the 2.3 fold difference in PM rates seen between lower and higher income countries [57].

Canada acknowledges that raising the health status of people with the greatest need would have a major impact on overall health and could also improve the nation’s productivity, as suggested by the WHO Commission’s report on health equity. Using a recently validated index of deprivation, our study demonstrates the feasibility of identifying and quantifying, at a small area-level, social and material factors that contribute to PM and health inequity. It is likely that the overall impact of social and material inequalities on health will continue to increase as the difference in wealth between the rich and the poor continues to grow [58]. Provincial and Federal governments in Canada and elsewhere have a responsibility to acknowledge and address these serious and growing issues that impact on health equity. Part of the effective delivery and evaluation of such policy changes must be the compilation of small area-level measures of health inequity and their determinants.

## Conclusion

In NS, approximately 32,000 people aged 15 years and older live in areas with extreme levels of deprivation, resulting in a doubling of their likelihood of dying prematurely. In this study, deprivation accounted for approximately 40% of premature deaths between 1995 and 2005, despite universal health care in Canada. The significant increases in PM with decreasing levels of social and material wealth observed in NS may reflect a small picture of what is happening at the national level and could translate into a serious public health burden. Also, while PM rates in those most privileged have been reported to be declining in recent years, those in the lower socioeconomic groups have either experienced slower proportional mortality decline or exhibited continued increase in PM. Part of this widening in health inequity may be due to a combination of individual characteristics and the environmental demands and constraints that affects the likelihood of adopting health-promoting behaviours. However, it could be argued that this growing inequity in health is rooted in greater societal inequities. Addressing the key factors that contribute to deprivation (e.g. employment, education, living arrangement), may suggest a form of intervention that would enable the individual to act on decisions that improve their health, which in turns would not only improve the health outcomes of Nova Scotians, but simultaneously reduce the health costs and burdens associated with an unnecessary and premature loss of life. Future studies should be designed to explore sex and age-specific patterns of socioeconomic deprivation on health. Analyses of age at death would allow the quantification of the number of potential years of life lost due to material and social deprivation. Based on a median age at death of 75 years, a person dying at 15 years of age results in the loss of 60 potential years of life, while that of a person aged 74 years results in the loss of only 1 potential years of life. Such quantification would allow the assessment of the absolute impact of socioeconomic disparity on health and provide a more focused profile of the global burden of health inequity.
